# The Soluble VEGF Receptor sFlt-1 Contributes to Impaired Neovascularization in Aged Mice

**DOI:** 10.14336/AD.2016.0920

**Published:** 2017-05-02

**Authors:** Guangxian Zhao, Xian W. Cheng, Limei Piao, Lina Hu, Yanna Lei, Guang Yang, Aiko Inoue, Shinyu Ogasawara, Hongxian Wu, Chang-Ning Hao, Kenji Okumura, Masafumi Kuzuya

**Affiliations:** ^1^Department of Cardiology, Yanbian University Hospital, Yanji, Jilin 133000, China; ^2^Department of Health Care & Geriatrics, Nagoya University Graduate School of Medicine, Aichiken 4668550, Japan; ^3^Department of Public Health, Guilin Medical College, Guilin, Guangxi 541004, China; ^4^Department of Cardiology, Shanghai General Hospital, Shanghai Jiao Tong University School of Medicine, Shanghai 20160527, China; ^5^Department of vascular surgery, Ren Ji Hospital, School of Medicine, Shanghai Jiao Tong University, Shanghai 200126, China; ^6^Department of Cardiology, Tohno Kosei Hospital, Mizunai, Japan; ^7^Institute for Future Society, NAGOYA STREAM, Nagoya University, Nagoya, Aichiken 4668550, Japan; ^8^Division of Cardiology, Department of Internal Medicine, Kyung Hee University, Seoul 130701, Republic of Korea

**Keywords:** aging, peripheral arterial disease, angiogenesis, soluble Flt1, Wnt5

## Abstract

The mechanism by which angiogenesis declines with aging is not fully understood. Soluble vascular endothelial growth factor receptor 1 (VEGFR1) form (sFlt1) contributes to endothelial dysfunction in pathological conditions. However, the roles of sFlt1 in ischemia-induced neovascularizationof aged animals have not been investigated. To study aging-related sFlt1 change and its impact on ischemia-induced neovascularization, a hindlimb ischemia model was applied to young and aged mice. Blood flow imaging assay revealed that the blood flow recovery remained impaired throughout the follow-up period. At day 14, immunostaining showed lesser capillary formation in the aged mice. An ELISA showed that the aged mice had increased plasma sFlt-1 levels at indicated time points after surgery. On operative day 4, the aged ischemic muscles had decreased levels of p-VEGFR2 and p-Akt and increased levels of sFlt-1, Wnt5a, and SC35 genes or/and protein as well as increased numbers of inflammatory cells (macrophages and leucocytes) and matrix metalloproteinase-9 activity. Immnunofluorescence showed that Flt-1 was co-localized with CD11b^+^ macrophages of aged ischemic muscles. Hypoxia stimulated sFlt1 expression in CD11b^+^ cells of aged bone-marrow (BM), and this effect was diminished by siWnt5a. The cultured medium of aged mice BM-derived CD11b^+^ cells suppressed human endothelial cell (EC) and endothelial progenitor cell (EPC) angiogenic actions induced by VEGF, and these decreases were improved by treatment with siWnt5a-conditioned medium. Thus, aging appears to decline neovascularization in response to ischemic stress via the VEGFR2/Akt signaling inactivation in ECs and ECPs that is mediated by Wnt5a/SC35 axis activated macrophages-derived sFlt1 production in advanced age.

Aging has been shown to impair the mammalian body’s ability to form new blood vessels under ischemic pathological conditions, which causes a diminished capacity for tissue regeneration [1]. In a wide range of mammals, the age-associated vascular failing and decline in revascularization are characterized by endothelial dysfunction and decreases in the numbers and intrinsic function of bone-marrow (BM)-derived endothelial progenitor cells (EPCs), a shift in the balance between vascular cell apoptosis and proliferation, and changes in the extracellular microenvironment (e.g., alterations in growth factors, inflammatory cytokines, and oxidative stress) [2-5]. Indeed, the molecular and cellular mechanisms responsible for the lack of a sufficient angiogenic response to chronic hypoxic stress in aged animals and humans remain largely unknown.

It is well known that biological hypoxia/ischemia usually cause increases in the expression of proangiogenic growth factors such as Vascular endothelial growth factor (VEGF) and its receptor (VEGFR) activation (including Flt1 and Flk1), which then initiate tubulogenesis from pre-existing vessels by inducing vascular cellular events (including proliferation, migration, and invasion) and vascular lumen maturation [6]. Accumulating evidence show that VEGF and VEGFR splicing can produce antiangiogenic actions under various pathological conditions [7-9]. The Soluble VEGFR1 (sFtl1), also known as a VEGF antagonist, is a splice variant of the VEGF receptor lacking the cytoplasmic and transmenbrane domains. Experimental and clinical studies have led to a number of important observations that contribute to our understanding of an inhibitory splice variant of sFlt1 [9-11]. For example, by binding and occupying the VEGFR, sFlt1 disturbs VEGF occupation and subsequent growth signal transduction in cultured cells [10]. It has been reported that sFlt1 resulted in endothelial dysfunction and angiogenic actions in the pathogenesis of preeclampsia [9]. Clinical study figured out to changes in plasma sFlt1 in metabolic disorder and atherosclerosis-based coronary artery disease that have relevance for the therapy and angiogenesis in these conditions [11]. A single recent vascular biological study demonstrated that Wnt5/SC35 activation contributed to impaired vascularization in peripheral artery disease in humans and animals [7]. Furthermore, a non-canonical Wnt-sFlt1 signal pathway has been shown to negatively regulate angiogenesis in myeloid cells [12].

The primary aim of our present study was to determine the distinct levels of sFlt1 and Wnt/SC35 axis in plasma and/or ischemic tissues between young and aged mice. We used the *in vitro* experimental strategies that to investigate the molecular mechanisms of the aging-related decline in the vascular regeneration capacity in aged mice with a special focus on macrophage Wnt/SC35-sFlt1 axis activation and endothelial cell (EC) VEGFR2/Akt signaling inactivation.

## MATERIALS AND METHODS

### Materials

Mouse anti-CD45 monoclonal antibody (mAb) (35-Z6, sc-1178), rabbit anti-Flt-1 polyclonal antibody (pAb) (C-17, sc-316), toll-like receptor 2 pAb (TLR2) (sc-10739), and goat GAPDH pAb (sc-20357) were purchased from Santa Cruz Biotechnology (Santa Cruz, CA). Flt-1 mAb (ab32152), VEGFR2 pAb (ab38473,), galectin-3 mAb (ab76245), and Wnt5a pAb (ab72583) were purchased from Abcam (Cambridge, MA). Rabbit Flk-1 pAb (#2472) and anti-rat IgG (Alexa Fluor 488 conjugate) were from Cell Signaling Technology (Danvers, MD). Rat anti-mouse Mac-3 (M3/84) mAb, SC35 mAb (aSC35), FITC-conjugated rat anti-mouse CD31 (MEC13.3) mAb, and R-PE-conjugated rat anti-mouse c-Kit (CD117, 2B8) mAb were purchased from BD Pharmingen (San Diego, CA). Rabbit anti-mouse IgG (Alexa Fluor 594 conjugate) was from Molecular Probes (Eugene, OR). siWnt5a (SASI_Mm01_00056405) and nontargeting control siRNA (#F5219292-009/010 as a negative control) were purchased from Sigma-Aldrich (Louis, MO). VEGF enzyme-linked immunosorbent assay (ELISA) Kits was purchased from R&D Systems (Munich, Germany). Endothelial basal medium (EBM)-2 and endothelial growth medium (EGM)-2 SingleQuotes were purchased from Lonza (Walkersville, MD). RPMI medium 1640 was from Life Technologies (Grand Island, NY). Human umbilical vein endothelial cells (HUVECs) were from Cell Applications (San Diego, CA). The β-Gal Staining Set and cOmplete Mini protease inhibitor cocktail were from Roche Diagnostics (Mannheim, Germany). RNeasy Micro Kits and SYBR™ Green Master Mix were from Qiagen (Hilden, Germany). Nitrocellulose transfer membrane was from Amersham Bioscience (Piscataway, NJ). Growth factor-reduced Matrigel Matrix was from BD Bioscience (Bedford, MA). Diff-Quik staining solution was from International Reagents Corp. (Kobe, Japan). The Cell Titer 96AO Assay kit was purchased from Promega (Madison, WI). The CD11b and CD117 microbeads kits and MACS separation columns was from Miltenyli Biotec (Bergisch-Gladbach, Germany). Lipofectamine Transfection reagent was from Invitrogen Life Technologies (Carlsbad, CA).

### Animals

All procedures that used animals were approved by the Institutional Animal Care and Use Committee of Nagoya University Graduate School of Medicine. The 2-month-old and >18-month-old C57BL/6J male mice (Chubukagakushizai, Nagoya, Japan) were provided with a standard diet and tap water *ad libitum*.

### Mouse hindlimb ischemic model and blood flow analysis

After being anesthetized with sodium pentobarbital (50 mg/kg intraperitoneally), male young and aged mice underwent left hindlimb ischemic surgery. In this model, the entire left vein and femoral artery were surgically removed [13]. We evaluated the blood flow recovery in the mice by laser speckle blood flow imaging (LSBFI, OMEGAZONE, OZ-1, OMEGA WAVE, Inc., Tokyo, Japan). An LSBFI evaluation was performed on the left and right legs and feet before surgery and on postoperative days 0, 4, 7, and 14. We used the changes in the laser frequency and different color pixels to express as the blood flow in each leg. The results of our quantitative analysis of leg blood flow are presented as the ratio of ischemic to non-ischemic LSBFI, in order to exclude data variations that are due to ambient temperature and light [14].

### Immunohistochemical analysis

On operative day 4 (for inflammation assays) or 14 (for capillary evaluation), we performed an immune-histochemical analysis using the antibodies Mac-3 (1:50) for macrophages, CD45 (1:40) for leukocytes, and CD31 (1:100) for capillary density on the ischemic and non-ischemic muscles of young and aged mice. We counted the numbers of leukocytes and macrophages in five random microscopic fields from four sections of each animal, and these values are expressed as the numbers of Mac-3- or CD45-positive cells per high magnification (×200). For the analysis of capillary density, we counted the capillaries and muscle fibers in four random microscopic fields from four different cross-sections of the adductor skeletal muscles in each animal, and these values are expressed as the number of capillaries per muscle fiber (×200) [15].

### Gene expression assays

Total RNA was isolated from the lysates (CD11b^+^ macrophages and c-Kit^+^ cells) and the muscle tissues with the use of RNeasy Micro Kits and then subjected to reverse transcription. The cDNA products were applied to a quantitative real-time polymerase reaction chain (PCR) analysis, as described [5]. Each targeted RNA level was normalized to its respective glyceraldehyde 3-phosphate dehydrogenase (GAPDH) mRNA level. Each sample was conducted in triplicate. The sequences specific to mouse (Wnt family and sFlt1) are summarized in Table 1.

**Table 1:** Primer sequences for mice used for quantitative real-time PCR

Gene name	Forward	Reverse
*mWnt3*	AGGAGTGCCAGCATCAGTTC	ACTTCCAGCCTTCTCCAGGT
*mWnt3b*	CTGGCAGCTGTGAAGTGAAG	TGGGTGAGGCCTCGTAGTAG
*mWnt4*	CTGGAGAAGTGTGGCTGTGA	CAGCCTCGTTGTTGTGAAGA
*mWnt5a*	CAAATAGGCAGCCGAGAGAC	CTCTAGCGTCCACGAACTCC
*mWnt5b*	CTGCTTGCGTAATGAGACCA	AAAGCAACACCAGTGGAACC
*mWnt7a*	GGTGCGAGCATCATCTGTAA	TCCTTCCCGAAGACAGTACG
*mWnt7b*	AAGCCTATGGAGACGGACCT	TTGGTGTACTGGTGCGTGTT
*mWnt8a*	AGCACAGAGGCTGAGCTGAT	TCTGCTCTCCTCTCCTCCAC
*mWnt9b*	TGGAGCGCTGTACTTGTGAC	GCACTTGCAGGTTGTTCTCA
*mWnt10a*	CATGAGTGCCAGCATCAGTT	ACCGCAAGCCTTCAGTTTAC
*mWnt10b*	GGAAGGGTAGTGGTGAGCAA	CTCTCCGAAGTCCATGTCGT
*mWnt11*	CAGGATCCCAAGCCAATAAA	GTAGCGGGTCTTGAGGTCAG
*msFlt1*	ATGCGTGCAGAGCCAGGAAC	GGTACAATCATTCCTCCTGC
*mGapdh*	TCACCACCATGGAGAAGGC	GCTAAGCAGTTGGTGGTGCA

### Gelatin zymography

Gelatin zymography was conducted as described [16]. Each sample was loaded onto a 10% polyacrylamide gel containing 1 mg/ml gelatin and 0.4% sodium dodecyl sulfate (SDS). After electrophoresis under nonreducing condition, the gels were cut down and washed with 2.5% Triton X-100 (v/v) twice for 30 min to remove the SDS and then incubated for overnight at 37°C with the reaction buffer (50 mM Tris-HCl, 0.15 M NaCl, 10 mM CaCl_2_, 0.02% NaN_3_, pH 7.4). The gels were then stained with 0.1% Coomassie Brilliant Blue, and the gelatinolytic activities of matrix metalloproteinase-2 (MMP-2) and MMP-9 appeared as clear bands against a blue-stained background.

### Immunoblotting assay

Proteins were extracted from muscle specimens by homogenization for 30 min in ice-cold lysis buffer (150 mM NaCl, 50 mM Tris-HCl, 1 mM EDTA, pH 7.4, 0.25% SDS, 1% Triton X-100)-supplemented protease inhibitor cocktail (1 tablet/10 mL), 1 mM phenylmethylsulfonyl fluoride adn1 mM sodium orthovanadate. Forty micrograms of proteins were loaded to electrophoresis in an SDS-polyacrylamide gel under reducing conditions and transferred to a nitrocellulose transfer membrane for testing with targeted primary antibodies, followed by the appropriate secondary antibodies. The densitometric analysis of the bands was conducted using ImageJ software.

### Cell culture

For the cell cultures, five-eight (young) and eighteen-twenty (old) population-doubling (PD) HUVECs were cultured in EBM-2 supplemented with 4% fetal bovine serum (FBS) and EGM-2 SingleQuotes in a humidified atmosphere of 95% air and 5% CO_2_. The PD was determined at each passage, as described [5]. β-galactosidase (β-gal) staining was applied to observe the HUVEC senescent phenotypes [5].

### Tubulogenesis assay

HUVECs at 2 × 10^4^ cells/well in a 24-well-plate were cultured for 24 hr on Matrigel in EBM-2 containing 20 ng/mL of VEGF to induce a tubulogenic response. For special experiments, the tubu formation was examined in the medium from young and aged mouse BM-derived c-Kit+ cells (called Yc-Kit^+^CM, Yc-Kit^+^CM) or CD11b^+^ cells (YCD11b^+^CM, ACD11b^+^CM) cultured under normoxic or hypoxic conditions at indicated concentrations for 24 hr (detailed in Figure Legend 5 and 6). Tubulogenesis was quantified using BZ-II analyzer, Exe 1.42 software to calculate the number and length of sprouts in six fields of each well[15].

### Cell migration, invasion, and proliferation assays

Cell migration and invasion assays were conducted using the Transwells of 24-well tissue culture plates, as described [13]. The cells that migrated and invaded the outer side of the membranes were stained using Diff-Quik staining solution and calculated in 5-7 chosen fields of the triplicate chambers for each sample at high magnification (×200).

The cell proliferation assay was performed using the Cell Titer 96AO Assay kit [15]. Cells were seeded on gelatin-coated 96-well plates at 5 × 10^3^ cells in 100 μL of EBM-2/0.3% bovine albumin serum (BAS) in the presence or absence of VEGF or one of four cell culture media (Yc-Kit^+^CM, Ac-Kit^+^CM, YCD11b^+^CM, and ACD11b^+^CM) at indicated concentrations for 48 hr. A 20-μL mixture of phenazine methosulfate and tetrazolium compound was directly added into the culture medium and incubated for 1 or 2 hr, and then absorbance was assayed at 492 nm. The values of each group in triplicate were averaged and are expressed as the absorbance’s intensity.

### ELISA

The sFlt1 levels in the plasma and cell-conditioned medium were determined using commercially available kits, as described [5].

### BM-derived EPC and macrophage isolation and culture

We collected BM-derived cells from both young and aged mice as described [17]. The c-Kit^+^ EPC-like cells and CD11b^+^ macrophages were isolated using MACS separation columns combined with CD117 microbeads or CD11b microbeads, respectively [17]. These cells were subjected to the related cell experiments.

### Immunofluorescence

After the adherence of BM-derived c-Kit positive cells (2 × 4^3^ cells/mL) to coverslips with denatured collagen, the cells were cultured in EGM-2 containing 4% FBS for 24 hr. Following fixation with 4% paraformaldehyde, the cells were washes with phosphate-buffered saline (PBS) containing 1% glycerol for 3 times. The cells were blocked with 0.1% BAS/PBS and then characterized using immunofluorescence [15]. Co-localization evaluations in ischemic muscles were conducted by double immunofluorescence staining. The frozen sections were pretreated with 3% BSA and treated with antibodies against sFlt-1 and CD11b (1:100 for overnight. Alexa Fluor 488-conjugated rabbit anti-mouse IgG and Fluor 594-conjugated chicken goat anti-mouse IgG (each 1:400) were used to visualize for immunoreactivity. The slides were mounted with glycerol-based Vectashied medium.

### siRNA transfection protocol

CD11b^+^ cells were grown on 6-mm dishes until they reached 80% confluence. The siRNA solution mixed with serum-free and antibiotic-free EBM-2 medium containing Lipofectamine^®^ Transfection reagent was added to each well to achieve a final siRNA concentration of 100 nM. The cells were incubated at 37°C for 48 hrs under hypoxic conditions (plates in hypoxic cambers), and the levels of targeted genes and proteins were analyzed by PCR and Western blotting assays. Transfected cells were also used for cellular functional experiments.

### Statistical analyses

All data are presented as the mean ± S.E.M in all analyses. Differences were compared by conducting a one-way analysis of variance (ANOVA) followed by Scheffe’s multiple-comparison post hoc test. A value of *P*<0.05 was considered significant. Two-way repeated-measures ANOVAs and Bonferroni’s post hoc tests were used for the statistical analysis of the blood flow and plasma sFlt1 data. The capillary density and length and number of endothelial sprouts were evaluated by two observers in a blind manner, and the values they obtained were averaged.

## RESULTS

### Aging impairs angiogenesis in response to ischemia

**Figure 1.**
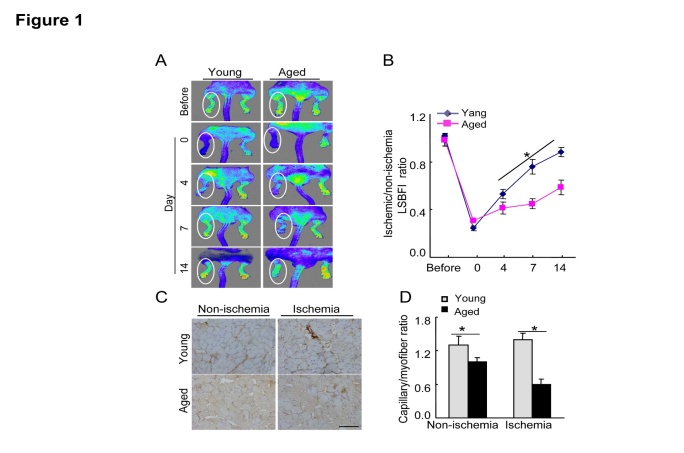
Aging impaired blood flow recovery and capillary formation in ischemic tissues **A)** Serial LSBFI showed that ischemic hindlimbs of the aged mice (>18 mos-old) exhibited a low perfusion signal (dark blue), whereas those of the young (2-mos-old) mice exhibited a high signal (red). **B)** The ratio of ischemia to normal LSBGI was lower in the aged mice compared to the young mice. **C)** On postoperative day 14, immunostaining was conducted to evaluate the capillaries in non-ischemic and ischemic thigh adductor muscles. **D)** Quantitative analyses revealed that aging reduced the capillary density in both non-ischemic and ischemic muscle compared to young mice. Data are mean ± SEM (*n*=5-6). *P<0.05 vs. corresponding controls (day 4, 7, and 14) by two-way repeated-measures ANOVA and Bonferroni *post hoc* tests or one-way ANOVA and Tukey’s *post hoc* tests. Scale bar, 50 μm.

We used a mouse hindlimb ischemia model to evaluate the impact of aging on ischemia-induced vascular regeneration. Our serial LSBFI analyses showed that the recovery of the ischemic/non-ischemic blood flow ratio in the aged mice remained impaired throughout the follow-up period (Fig 1A, B). On postoperative day 14, quantitative immunostaining revealed that the aged mice had lower capillary density in not only non-ischemic but also ischemic muscles compared to the young mice (Fig. 1C, D), suggesting that aging impairs vascular regenerative capacity.

**Figure 2.**
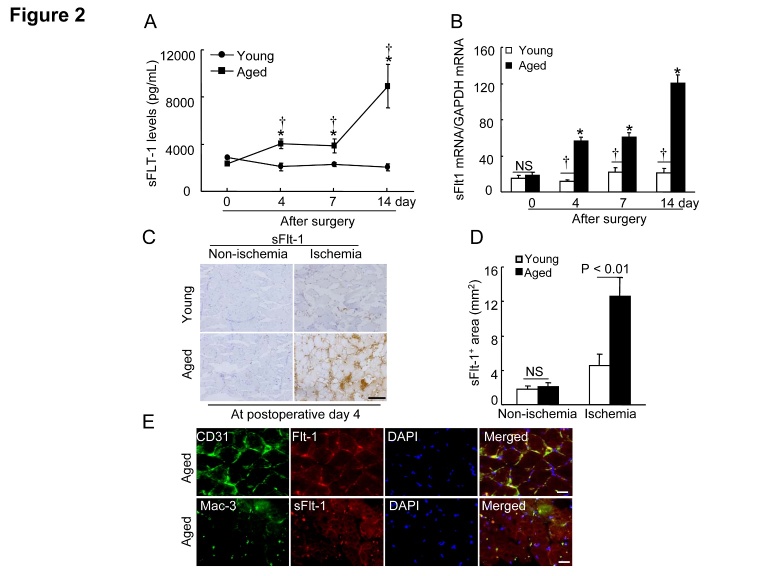
Aging increased the plasma and the ischemic muscle sFlt1 levels **A)** The ELISA showed that the aged mice had higher levels of circulating sFlt1 protein throughout the follow-up period (n=6-8). **B)** Quantitative real-time PCR showed that the aged mice had higher levels of sFlt1 gene throughout the follow-up period (n=6-8). Data are mean ± SEM. *P<0.05 vs. corresponding day 0; †*P*<0.05 vs. corresponding young mice during ischemia; two-way ANOVA and Bonferroni *post hoc* tests. **C** and **D**) Representative images and quantitative data shows that sFlt-1^+^ (including splice isoform sFlt-1 [77 kDa] and sFlt1 isoform 14 [82 kDa]) staining signal was markedly increased in the ischemic myofiber space of aged mice at postoperative day 4. **E**) Representative triple imunofluorescent images show that Flt-1 is expressed in endothelial cells as well as macrophages. Data are mean ± SEM (n=6). *P<0.05, †*P*<0.05 by one-way ANOVA and Tukey’s *post hoc* tests. NS indicates no significant. Scale bar, 50 μm.

### Impact of aging on sFlt1 expression and the downstream signaling pathway

**Figure 3.**
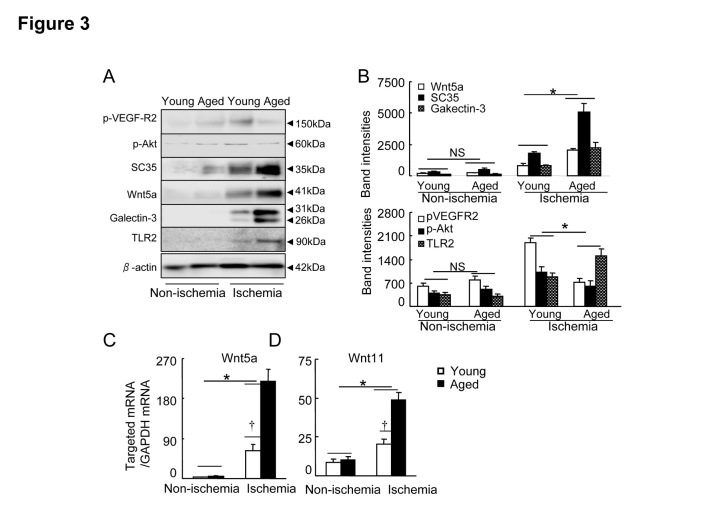
Levels of targeted protein in young and aged mice on postoperative day 4 **A)** Representative immunoblots show the levels of the targeted proteins in the ischemic and non-ischemic tissues of young and aged groups. **B)** Quantitative evaluation of Western blots for the levels of p-VEGFR2, p-Akt, SC35, Wnt5a, Galectin-3 and TLR2 proteins in young and aged mice (n=4). **C** and **D**) Quantitative real-time PCR showed the expression of Wnt5a (**C**) and Wnt11 (**D**) mRNAs (n=6). Data are mean ± SEM. *P<0.05, †*P*<0.05 by one-way ANOVA and Tukey’s *post hoc* tests.

It was reported that sFlt1 contributed to endothelial dysfunction and antiangiogenic responses in several pathological conditions [8, 9, 18]. As a first step to investigate whether aging affects on plasma sFlt1 level, we monitored the changes in the levels of plasma sFlt1 in young and aged mice (Fig. 2A). We also observed that aged ischemic muscles had dramatically increased sFlt1 gene levels compared to those of the young mice (Fig. 2B). Consistent with the gene expression assay, immunostaining analysis revealed that sFlt-1^+^ staining signal (i.e., splice isoforms sFlt-1 [77 kDa] and sFlt-1 14 [82 kDa]) was markedly increased in the ischemic myofiber space of aged mice (Fig. 2C and D), suggesting that aging can produce an antiangiogenic status. In addition, immunofluorescence show that Flt-1 is expressed in the endothelial cells as well as infiltrated macrophages (Fig. 2E). As shown in Figure 3A and B, the aged ischemic muscles had lower levels of p-VEGFR2 and p-Akt proteins compared to those of the young mice. Collectively, these observations suggest that the changes in the levels of sFlt1 expression might be responsible for the inactivation of VEGFR2/Akt, which is critical for the decline in ischemia-induced revascularization in aged animals.

### Impact of aging on Wnt5a/11 and SC35 expressions

Wnt5a activation has been shown to negative regulate ischemia-induced angiogenesis [7]. We therefore compared the levels of members of the Wnt family in the young and old mice. We analyzed the tissue extracts for several members of the Wnt family known to be involved in pathophysiological angiogenesis. Panels A and B of Figure 3 show that ischemic stress stimulated more Wnt5a protein expression in ischemic muscles of the aged mice compared to those of the young mice. Likewise, the aged ischemic muscles exhibited increased levels of Wnt5a mRNA (Fig. 3C). Similar to Wnt5a, the levels of Wnt11 gene were also higher in the aged mice (Fig. 3D). Wnt5a has been shown to upregulate SC35 in RAW24.7 cells [7]. As anticipated, the aged ischemic muscles had dramatically higher expression of SC35 protein compared to the young muscles (Fig. 3A, B). However, although ischemic stress stimulated the expression of targeted Wnt family members (including Wnt3, Wnt3a, Wnt5b, Wnt7a, Wnt7b, Wnt8a, Wnt9b, Wnt10a, and Wnt10b) in both young and aged mice, aging did not affect these members in non-ischemic and ischemic muscles (Table 2). These results indicated that aging can produce the changes in a non-canonical Wnt5a/SC35 axis levels in response to ischemia.

**Table 2:** Levels of targeted members of the Wnt family in ischemic and non-ischemic tissues of young and aged mice.

Parameter	Young nonischemia	Aged Nonischemia	Young Ischemia	Aged Ischemia
*mWnt3*	3.2 ± 1.4	5.6 ± 1.6	208.9 ± 43.2^*^	245.6 ± 53.8^*^
*mWnt3a*	4.3 ± 1.0	6.6 ± 1.8	298.7 ± 117.1^*^	216.1 ± 51.0^*^
*mWnt5b*	1.3 ± 0.3	1.8 ± 0.9	49.9 ± 14.8^*^	30.4 ± 4.3^*^
*mWnt7a*	6.5 ± 2.1	8.0 ± 2.0	213.0 ± 82.4^*^	164.1 ± 35.1^*^
*mWnt7b*	3.4 ± 1.3	7.0 ± 5.1	382.5 ± 106.0^*^	302.6 ± 58.1^*^
*mWnt8a*	3.2 ± 0.9	2.5 ± 0.7	23.2 ± 5.7^*^	20.7 ± 6.8^*^
*mWnt9b*	0.9 ± 0.3	1.1 ± 0.4	8.2 ± 2.1^*^	7.3 ± 3.1^*^
*mWnt10a*	13.8 ± 2.3	15.9 ± 2.5	45.7 ± 6.0^*^	49.1 ± 8.5^*^
*mWnt10b*	3.2 ± 1.7	1.6 ± 0.4	309.0 ± 60.1^*^	389.3 ± 107^*^

### Aging accelerates the inflammatory response in response to ischemia

**Figure 4.**
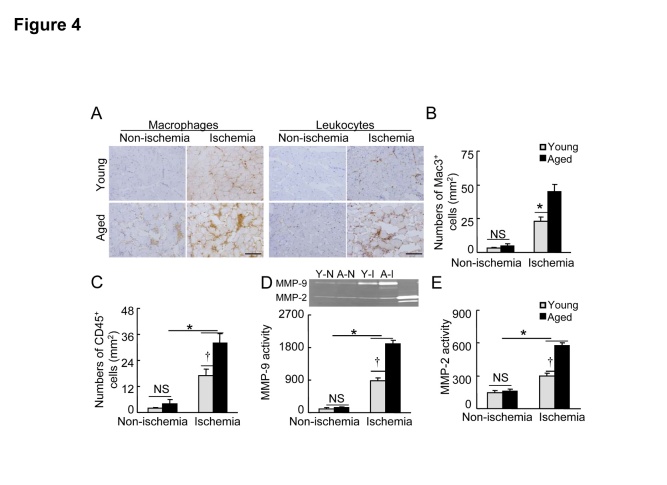
Aging accelerated the inflammatory cell infiltration in the ischemic muscles of the aged mice **A)** Immunostaining was conducted using antibodies against polymorphonuclear leukocytes (CD45) and macrophages (mac-3) in non-ischemic and ischemic tissues of the two groups on day 4 after surgery. Representative images (**A**) and combined quantitative data (**B, C**) showing the numbers of infiltrated mac-3^+^ and CD45^+^ cells (n=6). **D** and **E**) Representative gelatin zymography and combined quantitative data show MPP-9 and MMP-2 gelatinolytic activities in non-ischemic and ischemic tissues of both groups (n=4). Data are mean ± SEM. *P<0.05, †*P*<0.05 by one-way ANOVA and Tukey’s *post hoc* tests. Scale bar, 50 μm. NS indicates no significant.

Figure 3A and B shows that the levels of galactin-3 protein as well as TLR2 protein were increased in the ischemic muscles of aged mice compared with the young mice. Consistently, the present study’s immunochemical evaluation of ischemic and non-ischemic sections harvested on day 4 after surgery using mac-3 and CD45 antibodies revealed that higher numbers of leukocytes and macrophages were present in the extra-capillary space in ischemic muscles of the aged mice compared to those of the young mice (Fig. 4A, B), indicating that aging can induce excessive inflammatory responses in response to ischemic stress. In addition, we observed that the levels of the gelatinolytic activities for MMP-2 and MMP-9 were significantly increased in ischemic tissues of the aged mice compared to the young mice (Fig. 4C-E).

### Aging impairs progenitor cell intrinsic functions

**Figure 5.**
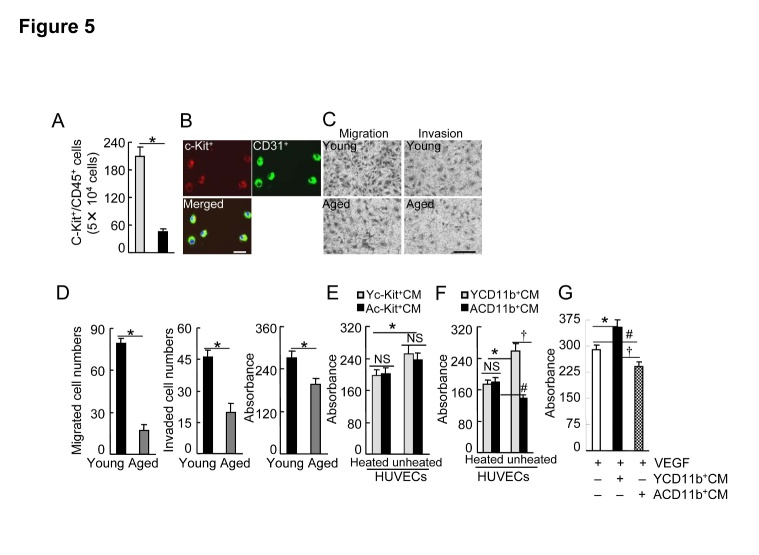
Effects of aging on BM EPC mobilization and cellular functions **A)** The flow cytometry analysis showed that the numbers of circulating c-Kit^+^/CD31^+^ cells were decreased in the aged mice compared to the young mice (n=6). **B)** Progenitor cell surface makers of (c-Kit and CD31) were expressed in BM-derived c-Kit^+^ cells cultured in EGM-2 for 4 days after isolation with magnetic beads. **C** and **D**) Representative images and combined quantitative data showing that aging impaired c-Kit^+^ cell migration, invasion, or/and proliferation (n=5-6). **E** and **F**) Yc-Kit^+^Cs or YCD11b^+^Cs and Ac-Kit^+^Cs or ACD11b^+^Cs were cultured (six-well-plates) in serum-free EBM-2 (for the former) or RPMI medium 1640 (for the later) under hypoxic condition (plates in hypoxic cambers) for 36 hr (for c-Kit+ cells) or 48 hr (CD11b+ cells), respectively. Following collection of the culture medium (Yc-Kit^+^CM, Ac-Kit^+^CM; YCD11b^+^CM, ACD11b^+^CM), the special fraction containing an approx. >70 kDa protein was isolated with 150 K and then 50 K AmiconUltra contricons, and we adjusted the protein concentration to 1.5 mg/ml for the cellular experiments. For the proliferation assays, 5 × 10^3^ HUVECs were incubated in EBM-2 supplemented with either heated or unheated Yc-Kit^+^CM, Ac-Kit^+^CM (**E**), YCD11b^+^CM, or ACD11b^+^CM (**F**) (20 μl/100 μl EBM-2 for each), respectively, for 48 hr, and then were subjected to MTS assays. **G)** HUVECs were incubated with VEGF-A (50 ng/mL), YCD11b^+^CM, or ACD11b^+^CM (20 μl/100 μl EBM-2 for both) respectively for 48 hr, and then were subjected to an MTS assay. Data are mean ± SEM (n=5-6). *P<0.05, †*P*<0.05, #*P*<0.05 by one-way ANOVA and Tukey’s *post hoc* tests. Scale bar, 50 μm.

Consistent with previous studies [4, 5, 19], we observed that the numbers of c-Kit^+^/CD31^+^ progenitor cells were decreased in the peripheral blood of the aged mice compared to the young mice (Fig. 5A). As impaired revascularization seems to be tightly associated with the decline in BM-derived EPC intrinsic functions, we isolated c-Kit^+^ using a magnetic method and extended our investigation of whether aging influences EPC cellular events induced by VEGF-A. As shown in Figure 5B, these cells expressed c-Kit and CD31 EPC surface markers. These results indicated that aging significantly impaired VEGF-A-induced c-Kit^+^ migration and invasion as well as proliferation (Fig. 5C and D).

### In the aged mice, BM-derived CD11^+^ cells exhibited antiangiogenic effects via the induction of sFlt1in response to hypoxia

We examined the effects of the c-Kit^+^ cells-conditioned culture medium under hypoxic condition on HUVEC proliferation. As shown in Figure 5E, unheated young c-Kit^+^ cells (Yc-Kit^+^Cs)-cultured medium (Yc-Kit^+^CM) and aged c-Kit^+^ cells (Ac-Kit^+^Cs)-cultured medium (Ac-Kit^+^CM) had comparable stimulator effects. We observed that unheated aged CD11b^+^ cell (ACD11b^+^Cs)-cultured medium (ACD11b^+^CM) impaired HUVEC proliferation compared to the heated medium, whereas unheated young CD11b^+^ cell (YCD11b^+^Cs)-cultured medium (YCD11b^+^CM) stimulated HUVEC proliferation (Fig. 5F). As shown in Figure 5G, YCD11b^+^CM enhanced VEGF-A-induced cell proliferation; this effect was abolished by replacement of the medium with ACD11b^+^CM.

**Figure 6.**
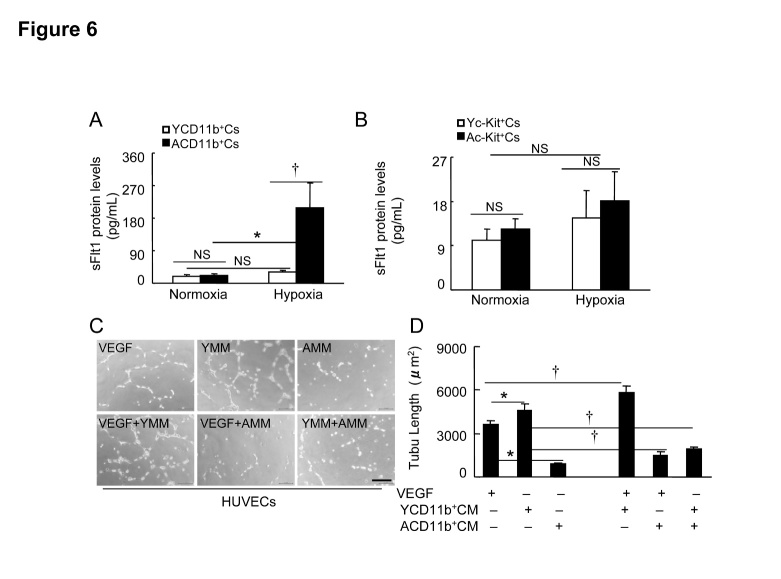
The effects of hypoxic stress on sFlt1 production in BM-derived c-Kit^+^ cells and CD11b^+^ cells of young and aged mice **A** and **B**) Subconfluent young mouse BM-derived c-Kit^+^ cells (Yc-Kit^+^Cs) or CD11b^+^ cells (YCD11b^+^Cs) and aged mouse BM-derived c-Kit^+^ cells (Ac-Kit^+^Cs) or CD11b^+^ cells (ACD11b^+^Cs) were cultured (six-well-plates) in serum-free EBM-2 (for the former) or RPMI medium 1640 (for the later) under hypoxic condition (plates in hypoxic cambers) for 36 hr (for c-Kit+ cells) or 48 hr (CD11b+ cells), respectively, and the conditioned media were then subjected to the ELISA with sFlt1 kits. **C** and **D**) Following collection of the culture medium (Yc-Kit^+^CM, Ac-Kit^+^CM; YCD11b^+^CM, ACD11b^+^CM) as the same as above, the special fraction containing an approx. >70 kDa protein was isolated with 150 K and then 50 K AmiconUltra contricons, and we adjusted the protein concentration to 1.5 mg/ml for the cellular experiments. HUVECs (2 × 10^4^) were cultured (24-well-plates) in EBM-2 containing VEGF-A, Yc-Kit^+^CM, Ac-Kit^+^CM, VEGF/Yc-Kit^+^CM, VEGF/Ac-Kit^+^CM, or Yc-Kit^+^CM/Ac-Kit^+^CM (20 ng/mL for VEGF-A; 90 μg/mL for the cultured media) respectively for 24 hr, and then subjected to length calculation. Representative images (**C**) and combined data (**D**) show the tubulogenesis in response to each stimulator. Data are mean ± SEM (n=6~8). *P<0.05 by one-way ANOVA and Tukey’s *post hoc* tests. Scale bar, 50 μm.

To explore these different effects, we determined the levels of the sFlt1 in four conditioned media. The ELISA results showed that BM-derived ACD11b^+^Cs had increased levels of sFlt1 protein in the conditioned medium in response to hypoxia compared to those of YCD11b^+^Cs (Fig. 6A). However, hypoxic stress had no effect on the sFlt1 protein production in the BM-derived Yc-Kit^+^Cs and Ac-Kit^+^Cs (Fig. 6B). YCD11b^+^CM stimulated HUVEC tubulogenesis compared to the VEGF positive control, whereas it was suppressed by ACD11b^+^CM (Fig. 6C, D). Likewise, YCD11b^+^CM accelerated the VEGF-induced HUVEC tubulogenesis, whereas the beneficial effects of VEGF-A and YCD11b^+^CM were abolished by the addition of ACD11b^+^CM. Taken together, these results suggest that BM-derived CD11b^+^ cells may be an important source of sFlt1 expression in response to hypoxic stress, contributing to the antiangiogenic response in aged mice.

### Up-regulation of Wnt5a/SC35 axis is responsible for the down-stream sFlt1 release and decreased VEGFR2/Akt-signaling-related EC and EPC angiogenic actions

**Figure 7.**
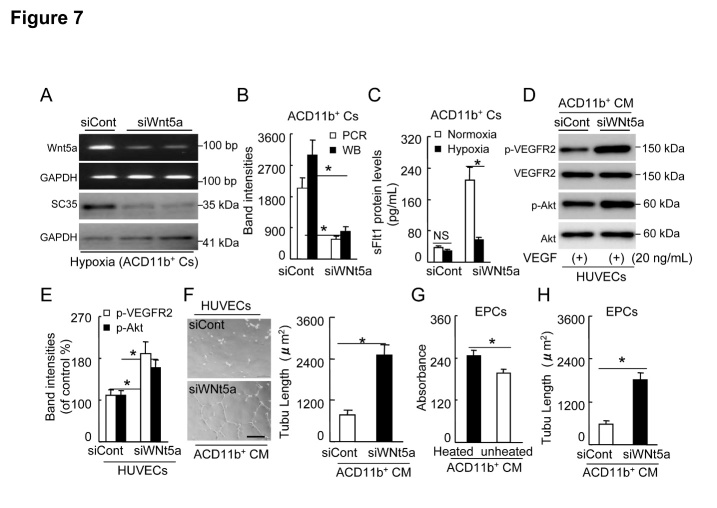
siWnt5a reduced the levels of Wnt5a mRNA and protein in aged mouse BM-derived ACD11b^+^Cs **A** and **B**) Subconfluent ACD11b^+^Cs were cultured (six-well-plates) in serum-free RPMI medium 1640 in presence of the siCont or siWnt5a under hypoxic condition for 24 hr, respectively, and the lysates were then subjected to the polymerase chain reaction (PCR) and the Western blotting (WB) assays. Representative PCR and immunoblots and combined quantitative data showed that siWnt5a decreased its mRNA and down-stream SC35 protein expression (n=3). **C**: Subconfluent ACD11b^+^Cs were cultured (six-well-plates) as the same conditions for 48 hr, respectively, and the conditioned media were then subjected to the ELISA with sFlt1 kits. The ELISA show that siWnt5a inhibited sFlt1 production in cultured ACD11b^+^Cs under hypoxia (n=8). **D** and **E**) Following collection of the culture medium of ACD11b^+^Cs treated as the same as above, the special fraction containing an approx. >70 kDa protein was isolated with 150 K and then 50 K AmiconUltra contricons, and we adjusted the protein concentration to 1.5 mg/ml for the cellular experiments. Representative immunoblots and combined quantitative data exhibit that siWnt5a-conditioned concentrated ACD11b^+^CM treatment (30 min) enhanced the enhancements of the VEGF-induced phospho-VEGFR2 (p-VEGFR2) and p-Akt as compared with siCont treatment in cultured HUEVCs (n=3). **F**) Representative images and combined quantitative data show that siWnt5a-conditioned ACD11b^+^CM ameliorated VEGF-induced HUVEC tubulogenic action as compared with control (n=6). **G**: As compared with unheated ACD11b^+^CM, unheated siWnt5a-conditioned ACD11b^+^CM exhibited an improvement of VEGF-induced young EPC-like c-Kit^+^ cell proliferation (n=7). **H**) siWnt5a-conditioned concentrated ACD11b^+^CM improved EPC-like c-Kit^+^ cell tubulogenesis (n=5). Data are mean ± SEM. *P<0.01 by one-way ANOVA and Tukey’s *post hoc* tests. NS indicates no significant. Scale bar, 50 μm.

As shown in Figure 7A-C, Wnt5a silencing suppressed not only its mRNA but also down-stream SC35 protein expression and sFlt1 release into culture medium of ACD11b^+^Cs under hypoxia, indicating that Wnt5a/SC35 axis is required for sFlt1 production in BM-derived ACD11b^+^Cs. In addition, as compared with siCont-conditioned concentrated ACD11b^+^CM, siWnt5a-conditioned concentrated ACD11b^+^CM markedly promoted the VEGF-induced phosphorylation of VEGFR2 and Akt in HUVECs (Fig. 7D and E). Likewise, siWnt5a-conditioned ACD11b^+^CM ameliorated HUVEC tubulogenic action in response to VEGF (Fig. 7F). On the other hand, unheated ACD11b^+^CM suppressed VEGF-induced young EPC-like c-Kit^+^ cell proliferation as compared with unheated ACD11b^+^CM (Fig. 7G).

Moreover, siWnt5a-conditioned ACD11b^+^CM exhibited a stimulatory effect on EPC tubulogenesis (Fig. 7H). Collectively, these observations suggest that Wnt5a/SC35 in activated aged macrophages may promote sFlt-1 release and subsequently suppresses VEGFR2/Akt signaling pathway to impair neovascularization capacity of aged mice.

## DISCUSSION

**Figure 8.**
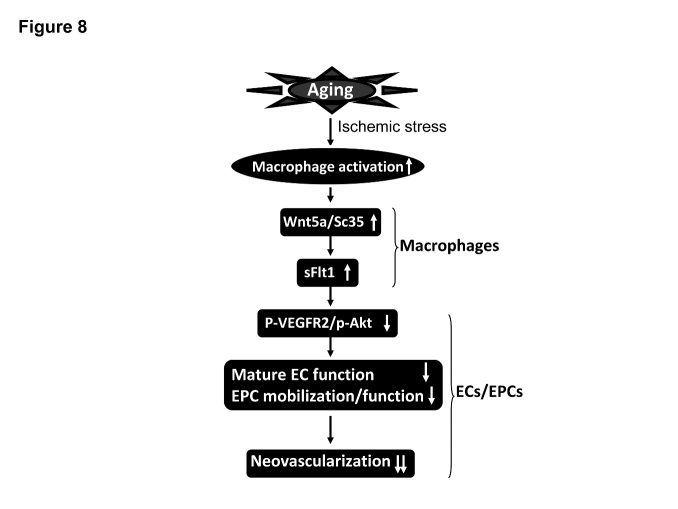
The proposed mechanism of the aging-related impairment of neovascularization in the mouse ischemic hindlimb model VGEFR2, vascular endothelial growth factor receptor 2 (VEGFR2), sFlt1, soluble Flt1, EC, endothelial cell; EPC, endothelial progenitor cell.

The present study made several significant findings as below: 1) Aging impaired ischemia-induced blood flow recovery accompanied with the increasing of plasma and ischemic muscle soluble Flt-1 levels; 2) The ischemic muscles of aged mice had decreased levels of p-VEGFR2 and p-Akt and increased levels of Wnt5a and SC35 expressions as well as increased numbers of infiltrated inflammatory cells and matrix metalloproteinase-9 activity; 3) Hypoxic stress stimulated sFlt1 expression in cultured ACD11b^+^Cs of aged BM, and this effect was reversed by siWnt5a treatment ; 4) The cultured medium of aged mouse BM-derived ACD11b^+^Cs under hypoxic conditions suppressed HUVEC and EPC angiogenic responses in response to VEGF; these effects were ameliorated by siWnt5a-conditioned ACD11b^+^CM. Thus, aging can impair neovascularization in response to hypoxia through the VEGFR2/Akt signaling inactivation in ECs and ECPs that is mediated by Wnt5a/SC35 activated macrophages-derived sFlt1 production in advanced age (Fig. 8).

Accumulating evidence show that sFlt1 induces endothelial dysfunction and anti-angiogenic responses in several pathological conditions [8, 9, 18]. It was reported that sFlt1 negatively regulates angiogenesis in myeloid cells[12]. In the present study, Aging impaired blood flow recovery and capillary density. The aged mice had higher plasma and ischemic muscle sFlt1 levels compared to the young mice throughout the follow-up period. Thus, aging appears to reduce revascularization in ischemic states through its ability to increase an antiangiogenic splicing isoform of sFlt1 production in response to ischemic stress. sFlt1 has been shown to inactivate downstream growth signaling [10]. Roberts and colleagues reported that Flt1 modulates VEGFR2 (Flk1) signaling during neovessel formation [20]. Our present findings show that the phosphorylations of VEGFR2 and Akt were decreased in the aged mice. Collectively, these findings suggest that sFlt1 may function as a key antiangiogenic regulator of ischemia-induced neovascularization in aged mice.

Previous *in vitro* experiments using myeloid cells showed that Wnt5 activation led to an enhancement of sFlt1 production [12]. Wnt5a signaling has been shown to be a noncanonical manner [21]. Our present findings show that ischemic stress increased the levels of Wnt5a gene in injured muscles of aged mice compared to young controls. Our protein expression assay showed that the levels of Wnt5a protein were also significantly increased in ischemic tissues of the aged mice compared to the young mice. In vitro experiments, siWnt5a suppressed sFlt1 production in BM-derived ACD11b^+^Cs in response to hypoxic stress. It was reported that a non-canonical Wnt5a suppressed angiogenesis in myeloid cells [12]. Thus, in aged mice Wnt5a activation appears to contribute to impaired revascularization in response to ischemia via the induction of sFlt1 production. Wnt5a can regulate SC35 expression in RAW264.7 cells [7]. Our Western blotting assay show that aging dramatically increased the SC35 expression in the ischemic tissues of the aged mice. Moreover, siWnt5a mitigates hypoxia-induced SC35 protein expression in activated ACD11b^+^Cs. Taken together, these data indicate that there is a close link between hypoxia-induced Wnt5a/SC35 axis activation and sFlt1 production in aged animals.

It has been shown that the function and the numbers of EPCs are reduced in animals and humans with cardiovascular risk factors such as aging [13, 19]. In our present study, the c-Kit^+^ cell migration, invasion and proliferation in response to VEGF were significantly impaired in the aged mice compared to the young mice. In addition, the numbers of c-Kit^+^/CD31^+^ cells were lower in the aged mice. Thus, the decline in the intrinsic function and the numbers of vascular progenitor cells could represent an explanatory mechanism in aging-related impairment of neovascularization to ischemia.

It was reported that inflammatory responses are accelerated by aging [13]. The exercise-mediated prevention of inflammatory responses to ischemia has been shown to ameliorate revascularization in aged animals [5]. Consistent with these studies [5, 13], we here observed that aged ischemic muscles had dramatically increased numbers of infiltrated macrophages and leukocytes compared to young muscles, suggesting that aging accelerates inflammatory over-responses to ischemic stress. This concept was further supported by previous [5, 13] findings and our current observation that the levels of infiltrated macrophage-derived MMP-9 gelatinolytic activity were higher in ischemic tissues of aged mice compared to those of young mice. In addition, our *in vitro* experiments demonstrated that hypoxia stimulated sFlt1 production in BM-derived CD11b^+^ macrophages. Unheated ACD11b^+^CM impaired HUVEC proliferation, whereas YCD11b^+^CM stimulated HUVEC proliferation. Our results showed that YCD11b^+^CM enhanced cell proliferation induced by VEGF-A; this effect was abolished by replacement of the medium with ACD11b^+^CM. YCD11b^+^CM caused HUVEC tubulogenesis and ACD11b^+^CM impaired it. Moreover, YCD11b^+^CM accelerated the VEGF-induced HUVEC tubulogenesis, whereas the VEGF-A- and YCD11b^+^CM-mediated benefits were abolished by the addition of ACD11b^+^CM. Thus, the macrophage activation-mediated enhancement of sFlt1 production could therefore represent a common mechanism in the antiangiogenic response to ischemic stress in aged animals. In addition, siWnt5a-conditioned concentrated ACD11b^+^CM markedly promoted the VEGF-induced phosphorylation of VEGFR2 and Akt in HUVECs. Likewise, siWnt5a-conditioned ACD11b^+^CM ameliorated VEGF-induced HUVEC tubulogenic action. On the other hand, unheated ACD11b^+^CM suppressed VEGF-induced young EPC proliferation. As anticipated, siWnt5a-conditioned ACD11b^+^CM exhibited a stimulatory effect on EPC tubulogenesis. Collectively, finding suggest that Wnt5a/SC35 in activated aged macrophages appear to be responsible for down-stream sFlt1 production and impaired VEGFR2/Akt signaling activation as well as the declined neovascularization capacity.

The results of the present study confirmed the dysfunctional hypoxia response in aged ischemic tissue with subsequent reductions in the levels of p-VEGFR2 and p-Akt proteins and enhancements of the levels ofsFlt1, Wnt5a, SC35, TLR2, galection-3, and MMP-9 genes or/and proteins and inflammation, offering a potential explanation for the disappointing consequence of clinical BM-derived cell therapies to date. Our findings suggest the macrophage Wnt5a/SC35-sFlt1 axis as a regulator of vascular response of ECs and EPCs to ischemic stress in the setting of aging-related cardiovascular disease, and they provide a novel potential molecular mechanism underlying the cross-link between aging and atherosclerosis-based cardiovascular disorders.
